# Working with Research Integrity—Guidance for Research Performing Organisations: The Bonn PRINTEGER Statement

**DOI:** 10.1007/s11948-018-0034-4

**Published:** 2018-05-31

**Authors:** Ellen-Marie Forsberg, Frank O. Anthun, Sharon Bailey, Giles Birchley, Henriette Bout, Carlo Casonato, Gloria González Fuster, Bert Heinrichs, Serge Horbach, Ingrid Skjæggestad Jacobsen, Jacques Janssen, Matthias Kaiser, Inge Lerouge, Barend van der Meulen, Sarah de Rijcke, Thomas Saretzki, Margit Sutrop, Marta Tazewell, Krista Varantola, Knut Jørgen Vie, Hub Zwart, Mira Zöller

**Affiliations:** 1 Work Research Institute, Oslo Met - Oslo Metropolitan University (Formerly Oslo and Akershus University College), Oslo, Norway; 2 The Norwegian Association of Researchers, Oslo, Norway; 3 EARMA - European Association for Research Managers & Administrators and University College Dublin, Dublin, Ireland; 4 Bristol Medical School: Population Health Sciences, University of Bristol, Bristol, UK; 5 Integrity Agency of the Municipality of Amsterdam, Amsterdam, The Netherlands; 6 University of Trento, Trento, Italy; 7 Law, Science, Technology & Society (LSTS), Vrije Universiteit Brussels, Brussels, Belgium; 8 Institute of Science and Ethics (IWE), University of Bonn, Bonn, Germany; 9 Institute for Science in Society, Radboud University, Nijmegen, The Netherlands; 10 Centre for Science and Technology Studies, Leiden University, Leiden, The Netherlands; 11 Oslo Metropolitan University (Formerly Oslo and Akershus University College), Oslo, Norway; 12 Radboud University Nijmegen, Nijmegen, The Netherlands; 13 Centre for the Study of the Sciences and the Humanities at University of Bergen, Bergen, Norway; 14 Research Coordination Office, KU Leuven, Leuven, Belgium; 15 Rathenau Instituut, The Hague, The Netherlands; 16 Institute of Political Science, Leuphana University of Lüneburg, Lüneburg, Germany; 17 Centre for Ethics, University of Tartu, Tartu, Estonia; 18 School of Social and Community Medicine, University of Bristol, Bristol, UK; 19 University of Tampere, Tampere, Finland; 20 Work Research Institute, OsloMet - Oslo Metropolitan University (Formerly Oslo and Akershus University College), Oslo, Norway; 21 Department of Philosophy and Science Studies, Faculty of Science, Institute for Science in Society (ISIS), Radboud University Nijmegen, Nijmegen, The Netherlands; 22 International Office, University of Bonn, Bonn, Germany

**Keywords:** Research integrity, Research misconduct, Organisational responsibilities, Guidance, Consensus conference

## Abstract

This document presents the Bonn PRINTEGER Consensus Statement: Working with Research Integrity—Guidance for research performing organisations. The aim of the statement is to complement existing instruments by focusing specifically on institutional responsibilities for strengthening integrity. It takes into account the daily challenges and organisational contexts of most researchers. The statement intends to make research integrity challenges recognisable from the work-floor perspective, providing concrete advice on organisational measures to strengthen integrity. The statement, which was concluded February 7th 2018, provides guidance on the following key issues:

1. Providing information about research integrity
2. Providing education, training and mentoring
3. Strengthening a research integrity culture
4. Facilitating open dialogue
5. Wise incentive management
6. Implementing quality assurance procedures
7. Improving the work environment and work satisfaction
8. Increasing transparency of misconduct cases
9. Opening up research
10. Implementing safe and effective whistle-blowing channels
11. Protecting the alleged perpetrators
12. Establishing a research integrity committee and appointing an ombudsperson
13. Making explicit the applicable standards for research integrity

## About the Document

Research integrity is inherently linked to the quality and excellence of research and science for policy. To further this agenda, the European PRINTEGER project (Promoting Integrity as an Integral Dimension of Excellence in Research) has conducted comprehensive studies on research integrity and misconduct.^1^ The research shows that there is a need for increased focus and guidance on how organisations may address such issues. In order to develop guidance that is anchored beyond the PRINTEGER project consortium, a consensus panel was established with a broader range of members representing wide practical and theoretical understandings of how to strengthen integrity in research organisations. The panel consists of members from different European countries and organisations, with diversity in terms of gender, geography, functions, seniority and disciplinary background.^2^ The members discussed recommendations in two rounds by email (a Delphi process) and at a final 1-day meeting during the PRINTEGER Conference on Research Integrity, in Bonn in Germany, February 7th 2018. This document presents the outcome of the consensus process.

The authors of this contribution are the signatories of the statement. While drawing on their professional backgrounds, the panel members are signatories of the statement in their private capacity. The statement represents the agreement of all members.

## Background

Research—and thus research misconduct—mostly takes place in a professional and organisational setting, and the organisations are normally held to be co-responsible for the conduct of their staff. There are therefore clear expectations (in some countries, legally mandated) for organisations to systematically work to promote responsible conduct in research, strengthen research integrity and reduce the risk of research misconduct. This document emphasises that responsibility for ethical research lies with everyone who is active in research, but especially with leaders in research performing organisations. Researchers’ morals alone cannot ensure research integrity; good conditions for exercising integrity must also be created at the level of the organisation and the research system.

There are a number of national, disciplinary and institutional codes and instruments that provide general guidance on research integrity. All European Academies (ALLEA) has issued the European Code of Conduct for Research Integrity.^3^ This is an important document outlining principles for research integrity, descriptions of good research practices and advice on how to deal with violations of research integrity. For instance, it suggests that research institutions and organisations should:

- Promote awareness and ensure a prevailing culture of research integrity;
- Demonstrate leadership in providing clear policies and procedures on good research practice and the transparent and proper handling of violations;
- Ensure that researchers receive rigorous training in research design, methodology and analysis;
- Develop appropriate and adequate training in ethics and research integrity and ensure that all concerned are made aware of the relevant codes and regulations.

To complement existing instruments, the current consensus statement focuses on institutional responsibilities for strengthening integrity. It takes into account the daily challenges and organisational contexts of most researchers. The statement intends to make research integrity challenges recognisable from the work-floor perspective, providing concrete advice on organisational measures to strengthen integrity. The consensus panel recognises that there is a broad range of integrity issues relevant to researchers (for instance related to corruption or sexual harassment), but focuses here only on research integrity.

The guidance is targeted at research leaders and managers at universities, colleges and public and private research institutes. Depending on their level in the hierarchy and the functions included in the role definitions, different leaders will have different responsibilities for taking action. In the guidance, we distinguish between recommendations to top level leaders (such as rectors or deans), middle level leaders (such as institute leaders) and project leaders. Some advice is also targeted to other actors in the research system; like the state and staff representative bodies. The recommendations may also prove relevant for leaders in other research-performing organisations, such as government research laboratories and industrial R&D units, as all research should be characterised by integrity.

Countries and organisations differ and organisations operate in different legal, economic and governance contexts. The consensus panel acknowledges these differences and their impact on how the organisations may work with research integrity, but believes that the recommendations presented in this document can be broadly applied.

The Consensus panel emphasises the following key issues:

1. Providing information about research integrity
2. Providing education, training and mentoring
3. Strengthening a research integrity culture
4. Facilitating open dialogue
5. Wise incentive management
6. Implementing quality assurance procedures
7. Improving the work environment and work satisfaction
8. Increasing transparency of misconduct cases
9. Opening up research
10. Implementing safe and effective whistle-blowing channels
11. Protecting the alleged perpetrators
12. Establishing a research integrity committee and appointing an ombudsperson
13. Making explicit the applicable standards for research integrity

## Guidance for Organisations’ Work with Research Integrity

### § 1. Providing Information About Research Integrity

Leaders at all levels of research-performing organisations should make sure that information about responsible research conduct is easily accessible and well-known among all employees, for instance through dissemination on the organisation’s webpages and intranets. This should include information about guidelines for research integrity, procedures in the case of observed misconduct, and relevant contact persons for more information. Dedicated persons should be made responsible for creating awareness of research integrity challenges, guidelines and procedures, and for ensuring that information is up-to-date and available for all. The ultimate responsibility for providing information lies with the institution, which should ensure a dedicated organisational support structure proportional to the size and complexity of the organisation.

While it is important for the organisation to focus on research integrity as a positive ideal, penalties for research misconduct must also be communicated and implemented.

### § 2. Providing Education, Training and Mentoring

Institutions are responsible for offering training and education to increase integrity and prevent misconduct, based on state-of-the-art knowledge. This should focus on good research and research management practices, and the risks of misconduct. They should be oriented towards situations researchers might realistically encounter at their different career levels and research contexts. Discipline-specific resources should be used when available and relevant. Training should be tailored to the institution, and provide the researcher with insight into the routines and tools that are available when one finds oneself in a difficult situation.

Inadequate mentoring and education of early career researchers is a risk factor for misconduct and supervisors bear a particular responsibility for the follow-up of early career researchers. However, education and training should be conducted at all levels, not only the Ph.D. level.

### § 3. Strengthening a Research Integrity Culture

Organizational culture is one of the most influential factors for individual conduct at workplaces; hence a strong integrity culture is a central prerequisite for responsible conduct in research. An integrity culture emphasises norms and values related to research integrity. Building a strong integrity culture involves sharing and giving substance to such norms, values and beliefs, emphasising their value for the organisation, attaching incentive structures to them and demonstrating good role models. It is the responsibility of top and middle management to set the standards for acceptable conduct and contribute to sharing good research practices. Leaders at all levels must themselves be good role models, and must strive for, and communicate clear expectations of, research integrity. In general, senior colleagues should contribute to the socialisation of more junior colleagues into a good integrity culture.

Short-term contracts, e.g., postdoctoral positions, may sometimes be unavoidable, but can be a barrier to longer term identification with the organisation and the knowledge of, and compliance with, the organisation’s values and ethical standards. In short-term, project-based positions, the role of the project leader in instilling ethical standards will be crucial, as staff on shorter contracts are often not integrated in the organisation to the same extent as permanent staff.

### § 4. Facilitating Open Dialogue

Researchers may come across new situations, where they inadvertently end up facing integrity challenges. Organizations must ensure that they create a safe and secure environment for researchers to identify and rectify mistakes and provide researchers with tools to make correct decisions, facilitating open discussion about dilemmas of research integrity. This will induce learning, both in the individual and in the organisations. There should also be room for challenging culturally embedded beliefs that may be counter-productive in an integrity perspective. Building bridges between different hierarchical levels by working toward a culture of open dialogue is an important action for strengthening integrity, as well as supporting transparency, fairness, collegiality and respect.

### § 5. Wise Incentive Management

Taking into account that indicators change the system through the incentives they establish, university leadership should adopt policies of good practice for responsible research assessment.^4^ As research-performing organisations may have various missions, strong incentives related to only one performance indicator, such as connecting bonuses to H-indexes or publication points assessments, may be counter-productive to research integrity. A broader set of key performance indicators and assessment approaches with a wider scope should be considered for assessment of individuals, groups and institutes, rather than certain specific metric outcomes that can induce misconduct or manipulation. National research policy makers should similarly be aware of potential effects of making university funding strongly dependent on a narrow range of indicators related to, for example, international peer-reviewed publications or patents, which may trickle down as pressures on individual integrity.

### § 6. Implementing Quality Assurance Procedures

By taking a positive interest in the work of the researchers, leaders can support and guide, and at the same time gain better information regarding actual research practices and practical dilemmas, allowing them to intervene if research integrity seems to be at risk. Leaders should establish and implement clear and transparent quality assurance procedures for all research. Research integrity should be an integral part of university strategies for improving the quality and impact of research.

### § 7. Improving the Work Environment and Work Satisfaction

A good work environment is associated with reduced risk of misconduct.^5^ If personnel perceive their work environment as good, and experience supportive follow-up from leaders and colleagues, this is conducive to research integrity. Continuous focus on creating a good work environment should be a natural part of research integrity efforts.

### § 8. Increasing Transparency of Misconduct Cases

In order to stimulate organisations’ capacity to learn from experience, there must be transparency. This means that organisations should be open about cases of confirmed research misconduct after they have been investigated, while safeguarding the legitimate rights to privacy and personal data protection of individuals, as regulated in national and European laws. The organisations should contribute to sharing practices and experiences in relevant fora.

Mandating organisations to report misconduct, and to cooperate with other organisations to collate this misconduct data, is likely to be effective in the long term. National policy makers should implement national reporting procedures so that organisations that openly report misconduct in good faith, do not find themselves penalised, while those institutions that cover up misconduct are not.

### § 9. Opening Up Research

The opening up of science to a broad range of researchers and stakeholders is becoming an increasingly important topic in research policy, and can function as a way to strengthen research integrity. Peer monitoring through open scholarly exchange should be facilitated by encouraging dissemination and sharing in the organisation and in other scholarly communities. Open science programs and frameworks for open data sharing can facilitate mutual monitoring among researchers, as well as research progress. Research leaders on all levels should strive to make their projects, preliminary results and final results available to the general public, potential users and the research community, in order to facilitate broader peer review and accountability of the research. This can be done by disseminating this information through project websites, the organisation’s own website or other established platforms for sharing of research. Data should be made available, potentially after a grace period of exclusive access for the organisation generating the data.

The need for confidentiality of any publically funded research should be explicitly justified. There are, however, legitimate reasons to restrict access, and the principle “as open as possible, as closed as necessary” can be a guide. In some cases, data sets must be kept confidential, for instance in order to protect the privacy of research subjects who may potentially be indirectly identified even if the personal data are anonymised.

### § 10. Implementing Safe and Effective Whistle-Blowing Channels

Alerting appropriate individuals to potential research misconduct can be done as part of regular reporting in the chain of command, but in some cases this can be difficult, for instance when leaders in the organisation are implicated. Other procedures must therefore be in place, more specifically, a whistle-blowing channel that is known, safe and works efficiently. A whistle-blowing channel may consist of a web-based mailbox or similar physical or online infrastructure where individuals may report allegations. This can be used by researchers and managers—or external collaborators—to notify appropriate individuals about alleged misconduct in the organisation. It can also be used to notify appropriate individuals about situations where researchers and managers themselves have experienced unacceptable practices (such as attempts to exert undue influence on the research from commissioners of the research or other stakeholders), which should be reported. The channel should have top-level support and attention, and should be monitored annually to assess the extent to which it is used and how users experience it. If found to be deficient, corrective actions must be taken to ensure its proper functioning. National policy makers should consider implementing regulations that allow relevant public authorities to follow-up on the adequacy of the procedures of whistle-blowing, and the handling of cases of alleged misconduct in the organisations.^6^

Whistle-blowers must be protected. The rights and duties of the whistle-blower must be clear and published on the organisation’s website. Whistle-blowers should have the option to be advised by a dedicated person before they bring forward an allegation.

### § 11. Protecting the Alleged Perpetrators

Researchers accused of misconduct are innocent until proven guilty. Their privacy must be protected throughout the whole investigation process in accordance with applicable legislation. In cases where accused researchers are cleared of accusations, appropriate measures must be taken to ensure that their names and reputations are not damaged or are repaired. As even groundless complaints may cause damage to a researcher, it should be made clear that malicious complaints are a breach of research integrity.

### § 12. Establishing a Research Integrity Committee and Appointing an Ombudsperson

There should be an integrity committee installed at the level of the institution or at the national level.

All research organisations should also have a research integrity ombudsperson. This function should be adequately resourced, well known in the organisation, and there should be a low threshold for contacting this person. Researchers who experience research integrity dilemmas or have come into an integrity related conflict should be able to discuss their case with the ombudsperson in a strictly confidential manner.

The function of the ombudsperson should be clearly separated from a formal research integrity committee, so it is clear to researchers that contacting the ombudsperson does not imply a formal registration of a case with the committee. The ombudsperson function could include the responsibility to continuously assess the research integrity status of the organisation, and advise on policies and action plans for strengthening the work on integrity.

### § 13. Making Explicit the Applicable Standards for Research Integrity

Researchers are often members of disciplinary professional organisations that have research integrity guidelines that may not be completely aligned with the organisational ones. They may also engage in multi-disciplinary, multi-organisational and multinational projects and networks where there are different standards for research integrity, for instance related to authorship.

Organisations should be aware of potentially conflicting standards and must have a policy for addressing them. Project leaders should seek to specify the standards the project will follow from the very beginning; most preferably by making this explicit in a collaboration agreement. The chosen standard must be well-justified and refer to generally accepted guidelines for research integrity. This collaboration agreement should also make explicit how allegations of research misconduct will be addressed in a multi-organisational project.^7^

## List of Signatories

- **Frank O. Anthun**, Special advisor at The Norwegian Association of Researchers, Norway.
- **Sharon Bailey**, Chair of EARMA - European Association for Research Managers and Administrators, and University College Dublin, Ireland.
- **Giles Birchley**, Senior Research Associate in Surgical Innovation and Bioethics, University of Bristol, United Kingdom.
- **Henriette Bout**, Senior advisor Integrity at the Integrity Agency of the municipality of Amsterdam (the Netherlands); Lecturer professional ethics and Philosophy of Science at the University of Amsterdam (the Netherlands); PhD candidate at the Nyenrode Business Universiteit (the Netherlands).
- **Carlo Casonato**, Professor of bio-law at the University of Trento, member of the National Bioethics Committee, Italy; founder and chief editor of the BioLaw Journal.
- **Ellen-Marie Forsberg**, Research professor, Head of Research at the Work Research Institute, Oslo Met - Oslo Metropolitan University (formerly Oslo and Akershus University College), Norway. [Secretariat]
- **Gloria González Fuster**, Research professor at Vrije Universiteit Brussels, Belgium.
- **Bert Heinrichs**, Professor of applied ethics, Institute of Science and Ethics (IWE), University of Bonn, Germany.
- **Serge Horbach**, PhD student, Institute for Science and Society, Radboud University, Nijmegen and Centre for science and technology studies, Leiden University, the Netherlands. [Secretariat]
- **Ingrid Skjæggestad Jacobsen**, Senior advisor and Secretary for the Research Ethics Committee at OsloMet - Oslo Metropolitan University (formerly Oslo and Akershus University College), Norway.
- **Jacques Janssen**, Confidential Advisor on Academic Integrity Radboud University, Professor Emeritus Psychology of Culture and Religion, Radboud University Nijmegen, the Netherlands.
- **Matthias Kaiser**, Professor, Director of the Centre for the Study of the Sciences and the Humanities at University of Bergen, Norway. [Secretariat]
- **Inge Lerouge**, Research Integrity & Ethics Coordinator at the Research Coordination Office Unit, KU Leuven, Belgium.
- **Barend van der Meulen**, Head of Research at the Rathenau Instituut, Professor Evidence for Science Policy at Leiden University, and chair of the Scientific Integrity Committee of Wageningen University & Research, The Netherlands.
- **Sarah de Rijcke**, Professor of Science and Evaluation Studies, Centre for Science and Technology Studies, Leiden University, the Netherlands.
- **Thomas Saretzki**, University Professor for Political Theory and Public Policy at Leuphana University of Lüneburg, Germany.
- **Margit Sutrop**, Practical Philosophy, Head of the Centre for Ethics, Dean of the Faculty of Arts and Humanities at the University of Tartu, Estonia.
- **Marta Tazewell**, Research Associate for CAP and ProtecT Trials Data Management, University of Bristol, United Kingdom.
- **Krista Varantola**, Professor emerita University of Tampere; Board member of ALLEA (All European Academies) and Member of the ALLEA Permanent Working Group on Science and Ethics; Chair of the National Board on Research Integrity, Finland.
- **Knut Jørgen Vie**, PhD student PRINTEGER project, Work Research Institute, OsloMet - Oslo Metropolitan University (formerly Oslo and Akershus University College), Norway. [Secretariat]
- **Mira Zöller**, International Office, University of Bonn, Germany. [Secretariat]
- **Hub Zwart**, Professor; Director of Education at the Institute for Science in Society (ISIS) (Director of Education); Chair of the Department of Philosophy and Science Studies (Chair); Scientific director of the Centre for Society and the Life Sciences (CSG); Coordinator of the PRINTEGER project; all at Radboud University Nijmegen, the Netherlands. [Secretariat]

## Footnotes

1 www.printeger.eu.

2 See short bios in List of Signatories.

3 http://ec.europa.eu/research/participants/data/ref/h2020/other/hi/h2020-ethics_code-of-conduct_en.pdf.

4 See the Leiden Manifesto: http://www.nature.com/news/bibliometrics-the-leiden-manifesto-for-research-metrics-1.17351.

5 Ref. PRINTEGER survey deliverable: http://printeger.eu/wp-content/uploads/2018/02/D4.2.pdf.

6 As an example, in Finland all public research organisations need to sign the national code of conduct TENK “Responsible conduct of research and procedures for handling allegations of misconduct in Finland”, http://www.tenk.fi/en/responsible-conduct-of-research.

7 See the Organisation for Economic Co-operation and Development (OECD) boilerplate text: https://www.oecd.org/sti/sci-tech/42770261.pdf.

